# Characterization of *WOX* genes revealed drought tolerance, callus induction, and tissue regeneration in *Gossypium hirsutum*

**DOI:** 10.3389/fgene.2022.928055

**Published:** 2022-10-12

**Authors:** Sani Muhammad Tajo, Zhaoe Pan, Shoupu He, Baojun Chen, Yusuf KM, Tahir Mahmood, Salisu Bello Sadau, Muhammad Shahid Iqbal, Teame Gereziher, Umar Suleiman Abubakar, Masha Joseph, Tajo Sammani, Xiaoli Geng, Xiongming Du

**Affiliations:** ^1^ State Key Laboratory of Cotton Biology, Institute of Cotton Research, Chinese Academy of Agricultural Science, Anyang, China; ^2^ Bioresources Development Centre, National Biotechnology Development Agency, Abuja, Nigeria; ^3^ Department of Agricultural Economics, University of Maiduguri, Maiduguri, Nigeria

**Keywords:** cotton, WUSCHEL-related Homeobox genes, drought, regeneration, expression analysis, virus-induced gene silencing

## Abstract

Cotton is an important natural fiber crop; its seeds are the main oil source. Abiotic stresses cause a significant decline in its production. The WUSCHEL-related Homeobox (WOX) genes have been involved in plant growth, development, and stress responses. However, the functions of *WOX* genes are less known in cotton. This study identified 39, 40, 21, and 20 *WOX* genes in *Gossypium hirsutum*, *Gossypium barbadense*, *Gossypium arboreum*, and *Gossypium raimondii*, respectively. All the *WOX* genes in four cotton species could be classified into three clades, which is consistent with previous research. The gene structure and conserved domain of all *WOX* genes were analyzed. The expressions of *WOX* genes in germinating hypocotyls and callus were characterized, and it was found that most genes were up-regulated. One candidate gene *Gh_ A01G127500* was selected to perform the virus-induced gene silencing (VIGS) experiment, and it was found that the growth of the silenced plant (*pCLCrVA: GhWOX4_A01*) was significantly inhibited compared with the wild type. In the silenced plant, there is an increase in antioxidant activities and a decrease in oxidant activities compared with the control plant. In physiological analysis, the relative electrolyte leakage level and the excised leaf water loss of the infected plant were increased. Still, both the relative leaf water content and the chlorophyll content were decreased. This study proved that *WOX* genes play important roles in drought stress and callus induction, but more work must be performed to address the molecular functions of *WOX* genes.

## Introduction

Cotton is the world’s most important fiber and oil crop (Campbell et al., 2010; Horiguchi et al., 2012). The upland cotton (*Gossypium hirsutum* L.) provides 35% of the fiber used worldwide (Abdelraheem et al., 2019). Cotton seed oil accounts for around 16% of the total weight of the seed (Liu et al., 2009). With global climate change, more and more environmental problems are threatening the growth and development of cotton, such as drought, salinization, and warming. Drought and heat stress have severely affected cotton production, leading to about 34% fiber yield loss (Ullah et al., 2017). Multiple interacting genes control drought tolerance to induce a morphological and physiological response, including cell membrane stability, chlorophyll levels, and relative water content (Gadallah, 1995). The development of stress-tolerance cotton is of great significance in sustaining world agriculture production. Drought stress-responsive genes comprise many groups based on their biological functions. Among these groups, transcription factors were more important because of their potential to regulate numerous downstream genes, such as *DREB2*, *SNAC1*, *ABF*, *OsSIZ1*, and *AVP1* (Zhang et al., 2010; Liu et al., 2014; Kerr et al., 2018; El-Esawi and Alayafi, 2019).

WUSCHEL-related Homeobox (WOX) gene family is a plant-specific homeobox (HB) transcription factor family with a short stretch of amino acids (60–66 residues) that forms a DNA-binding domain known as homeodomain (Graaff et al., 2009). The homeodomain of WOX protein binds to DNA through a helix-turn-helix structure characterized by two α-helices and a short turn. Phylogenic analysis of WOX proteins in multiple higher plant species, including *Arabidopsis*, rice, soybean, and maize, showed that the *WOX* gene family could be classified into three clades which include the ancient clade, intermediate clade, and WUS clade (Kamiya et al., 2003; Haecker et al., 2004; Nardmann and Werr, 2006; Hao et al., 2019). Previous studies showed that *WOX* genes have important roles in many aspects of growth and development, including embryonic development and polarization, meristematic stem cell maintenance, later organ development, seed formation, and regeneration (Park et al., 2005; Liu et al., 2009; Shimizu et al., 2009; Zhang et al., 2010; Zhang et al., 2011; Chu et al., 2013; Xu et al., 2015; Segatto et al., 2016). In *Arabidopsis thaliana*, the *WOX* gene family comprises 15 members that play similar roles in the initiation and/or maintenance of diverse embryonic, meristematic cells, and organs (Haecker et al., 2004). *AtWUS* is necessary for stem apical meristem formation and maintenance (Gross-Hardt et al., 2002). In rice, the *OsWOX4* gene regulates a number of pathways, including phytohormone signaling and cell development (Yasui et al., 2018). Overexpression of *GmWOX18* increased the regeneration ability of clustered buds (Hao et al., 2019). *VvWOX* genes appeared to be key regulators of somatic embryogenesis in grapevine (Gambino et al., 2011). Agrobacterium-mediated genetic transformation of cotton was described in 1980s but is still time-consuming and genotype-dependent due to poor regeneration. Overexpression of *AtWUS* promoted somatic embryogenesis and induced organogenesis in cotton (Bouchabke-Coussa et al., 2013). Overexpression of *GhWUS* in *Arabidopsis* promoted shoot regeneration from the excised root without exogenous hormones (Xiao et al., 2018). In addition to the function of *WOX* genes in plant development and regeneration, some genes play important roles in abiotic stress. *AtWOX6*, also known as *HOS9-1*, plays an important role in freezing tolerance independent of the C-repeat binding factor pathway (Zhu et al., 2004). Overexpression of *OsWOX13* in rice resulted in drought resistance and early flowering (Minh-Thuet al., 2018). Most of the *WOX* genes from rice were responsive to drought, salt, and cold treatment (Cheng et al., 2014). Most of the *WOX* genes in soybean responded to cold and drought stress treatments (Hao et al., 2019).

The roles of the *WOX* gene family have been well documented in *Arabidopsis*, maize, and rice. However, the functions of *WOX* genes in callus induction, regeneration, and abiotic stress are largely unknown in cotton. This study aimed to identify the *WOX* genes in cotton based on updated genome sequences of four species, examine their gene structure and expression profiles, and characterize their molecular roles in response to drought stress and callus induction.

## Materials and methods

### Plant material and germination

Three varieties of *G. hirsutum* with higher regeneration ability including Zhongmainsuo24 (ZM24), Coker312 (C312), and YZ-1, and one cultivar Texas Marker-1 (TM-1) with lower regeneration ability were selected based on previous reports (Jin et al., 2006; Zheng et al., 2014; Cao et al., 2017; Chen et al., 2022). All these seeds were obtained from the Mid-term GeneBank of the Institute of Cotton Research of Chinese Academy of Agricultural Science. Seeds were delinted and disinfected with 0.1% HgCl_2_ (W/V) by blending for 10 min, and then washed five times with sterilized distilled water for 2 min each time. To induce hypocotyl germination, 30 healthy seeds were placed on a sterilized filter paper in a petri plate, cultured in sterilized distilled water, and stored in a dark chamber for 48 h at 28°C (Kumar et al., 2015).

### *WOX* gene characterization in *Gossypium* species

To identify cotton *WOX* genes, the full-length sequences of 15 *AtWOX* genes which were downloaded from the *Arabidopsis* genome database (https://www.arabidopsis.org) were used as queries for BLASTp in CottonFGD (www.cottonfgd.net). We further confirmed all *WOX* genes in cotton using Pfam (http://pfam.xfa.org). Protein length, weight, charge, isoelectric point (pI), and GRAVY were available from CottonFGD (Supplementary Table S1).

### Chromosomal mapping, phylogenetic tree, gene structure, and conserved motif analysis of *WOX* genes

We used the GFF3 dataset from CottonFGD and gene IDs to assess the distribution of *WOX* genes across all chromosomes of *G. hirsutum*, *G. barbadense*, *G. raimondii*, and *G. arboreum*, and the result was visualized using the TBtools (Chen et al., 2020). ClustalX was utilized to perform multiple sequence alignment (Larkin et al., 2007). The neighbor-joining (NJ) strategy was used to decide the advancement distance with 1000 bootstrap replications by MEGA 5.0 (Tamura et al., 2011). The *WOX* gene structure was analyzed using the Gene Structure Display Server 2.0 (http://gsds.cbi.pku.edu.cn/). MEME (http://meme-suite.org) was used to discover the conserved motifs.

### RNA sequencing data analysis

*WOX* gene IDs from *G. hirsutum* were retrieved from CottonFGD, and corresponding gene IDs from variety ZM24 were retrieved from GRAND (http://grand.cricaas.com.cn/home). Heml software was used to demonstrate the expression (Deng et al., 2014).

### Medium preparation and callus induction

Seven-day-old seedlings have been divided into hypocotyl, cotyledon, and shoot tip. Individual hypocotyl and cotyledon were transplanted into Murashige and Skoog (MS) medium containing 2,4-dichlorophenoxyacetic acid (2,4-D, 0.5 mg/L) and kinetin (0.1 mg/L), and shoot tips were transplanted into MS medium containing 2,4-D (0.5 mg/L) and kinetin (0.2 mg/L) (Jin et al., 2006).

### RNA extraction and qRT-PCR analysis

Total RNA was extracted from hypocotyls and callus using RNAprep Pure Plant Plus Kit (TIANGEN, Beijing, China), following the manufacturer’s instructions. RNA concentration and purity were measured using NanoDrop 2000. The RNA was reverse-transcribed to cDNA by using the transcript Reverse Transcriptase (TransGen, Beijing, China). The specific primers of *WOX* genes for qRT-PCR are listed in Supplementary Table S2. The ABI 7500 Fast Real-Time System (Applied Biosystems, Foster City, CA, United States) was used for the qRT-PCR experiment. Each reaction included 1 µL of cDNA, 2 µL of forward and reverse primers, 6 µL of RNA-free water, and 10 µL of SYBR solution. *GhActin* was employed as an internal control in three biological and technical replications. Gene expression levels were calculated using the 2^−ΔΔCt^ method (Livak and Schmittgen, 2001).

### Protein interaction network prediction

Interaction network analysis of WOX proteins was performed with STRING with default parameters (version 11.0, https://string-db.org/cgi/input.pl) on the foundation of the homologous proteins in *Arabidopsis* (Szklarczyk et al., 2019).

### Transactivation activity assay

The GAL4 DNA-binding domain was fused with the cotton *GhWOX4_A01* gene into pGBKT7. pGBKT7-*GhWOX4_A01*, pGBKT7, and positive control pGADT7-largeT + pGBKT7-p53 were transformed into the AH109 yeast by utilizing the Clontech technique to investigate auto-activation and toxicity. The transformed yeast cells were cultured on SD/-Trp, SD/-Trp + X-α-gal, or SD/-Trp/-His/-Ade media and incubated at 30°C for 3–5 days.

### Virus-induced gene silencing of *GhWOX4_A01* and drought treatment

A 339-bp fragment of *GhWOX4_A01* was amplified from the cDNA of TM-1 by using gene-specific forward and reverse primers to construct a VIGS vector (Supplementary Table S2). The PCR product was then digested with *Spe* I and *Acs* I, and cloned into *Spe I-Acs I -Cut pCLCrVA*. The fusion vector was named *pCLCrVA: GhWOX4_A01* and transformed into *Agrobacterium tumefaciens* strain LBA4404. The control vector *pCLCrVA*, *pCLCrVA: GhWOX4_A01* and positive vector *pCLCrVA: PDS* were mixed with *pCLCrVB* at a 1:1 ratio (Gu et al., 2014). The mixed *Agrobacterium tumefaciens* solutions were injected into the two-week-old cotton cotyledons of *G. hirsutum* variety H177 on the abaxial side with a needle-free syringe. The plants were placed at room temperature in the dark overnight and grew at 23°C with a 16 h/8 h light/dark cycle. The study on *Agrobacterium* infection was carried out three times with 30 plants for each vector. After 4 weeks, the plants injected with empty control *pCLCrVA* and *pCLCrVA: GhWOX4_A01* were subjected to drought treatment. For drought treatments, plants were irrigated with 15% PEG6000 (w/v) for 2 weeks to detect their drought response, while control plants were irrigated with 1/2 MS nutrient solution. Under control and drought circumstances, the concentrations of antioxidant enzymes catalase (CAT) and peroxidase (POD), and oxidants malondialdehyde (MDA) and H_2_O_2_ were determined in both control and silenced plants. After drought treatment, important parameters were investigated, including ion leakage, excised leaf water loss, chlorophyll concentration, and relative leaf water content. The experiments were performed with three independent biological repetitions.

### Statistical analysis

GraphPad Prism (version 8.4.3; GraphPad Software, La Jolla California, United States) was used to analyze the quantitative data generated from the experiments. All the experiments were carried out with three replications. Analysis of variance (ANOVA) and multiple comparisons (Fisher’s LSD) were used to investigate the statistical significance of different treatments. The significance level for the different treatments was labeled as different letters under *p* < 0.05.

## Result

### Sequence analysis and chromosome mapping of *WOX* genes identified in four cotton species

We have identified 39, 40, 21, and 20 *WOX* (WUSCHEL-related Homeobox) genes in *G. hirsutum*, *G. barbadense*, *G. arboreum*, and *G. raimondii*, respectively (Supplementary Table S1). In *G. hirsutum*, the amino acid length of WOX proteins stretches from 125 aa to 383 aa, and the molecular weight ranges from 14.10 to 42.90 kDa. The amino acid length of *WOX* proteins in *G. barbadense* ranged from 187 aa to 369 aa, and the molecular weight ranged from 21.78 to 41.93 kDa. In *G. arboreum*, the amino acids stretch from 77 aa to 364 aa, and molecular weight ranges from 9.17 to 41.77 kDa. In *G. raimondii*, the amino acids stretch from 188 aa to 377 aa, and their molecular weight is within the range of 21.89–41.73 kDa. All the WOX proteins in the four cotton species have negative GRAVY values, indicating that all proteins were hydrophilic.

**FIGURE 1:**
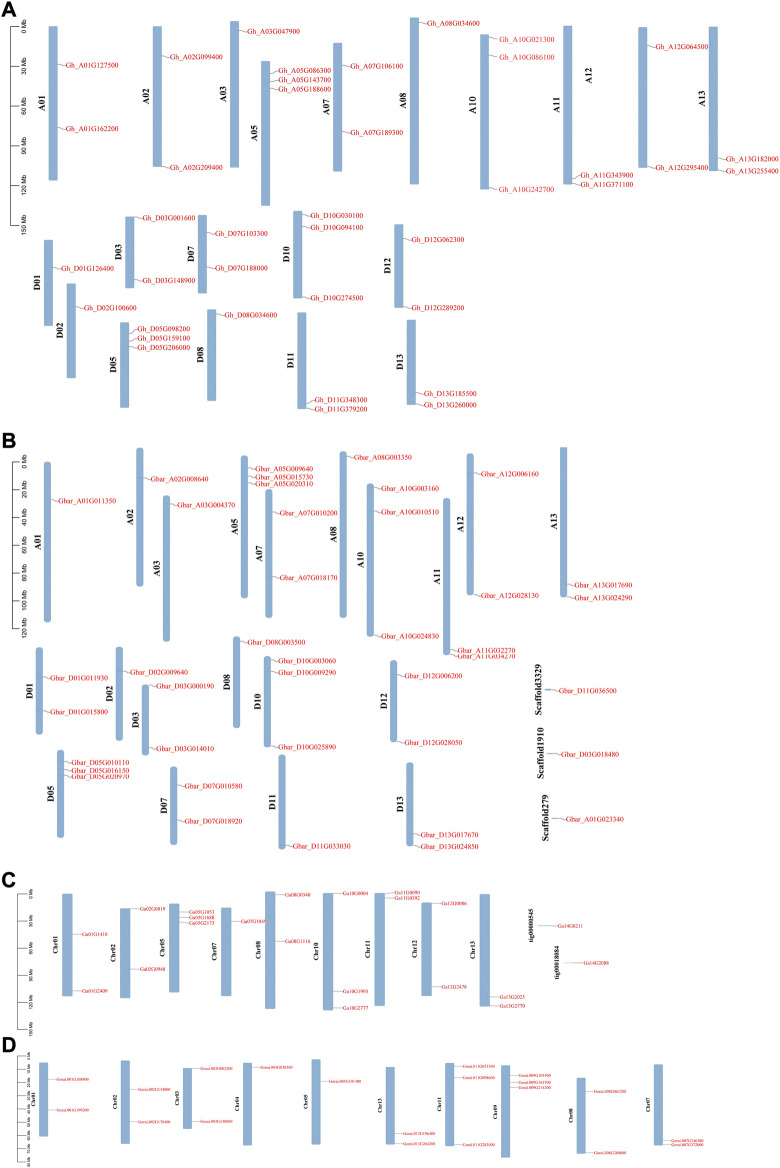
*WOX* genes position on the chromosome in *Gossypium* species. **(A)** *G. hirsutum*, **(B)** *G. barbadense*, **(C)** *G. arboreum*, and **(D)** *G. raimondii*.

*WOX* genes were not evenly distributed across the chromosomes in the four cotton species (Figure 1). In *G. hirsutum* and *G. barbadense*, chromosomes A05, D05, A10, and D10 possessed the highest number of *WOX* genes (3). In *G. hirsutum*, *WOX* genes were missing on chromosomes A04, D04, A06, D06, A09, and D09. Similarly, *WOX* genes were also missing on chromosomes A04, D04, A06, D06, A09, and D03 in *G. barbadense*. In *G. arboreum*, chr05 and chr10 had most of the genes (3). *WOX* genes were missing on chr03, chr04, chr06, and chr09 but had two genes on scaffolds in *G. arboreum*. In *G. raimondii*, chr09 and chr11 had the highest number of genes (3). *WOX* genes were missing on chr06 and chr10 in *G. raimondii.* The total number of *WOX* genes identified in the two diploid species was higher than that in the tetraploid *G. hirsutum* due to the hybridization of progenitors resembling *G. arboreum* and *G. raimondii*.

### Phylogenetic tree, gene structure, and conserved domain analysis of *WOX* genes

**FIGURE 2:**
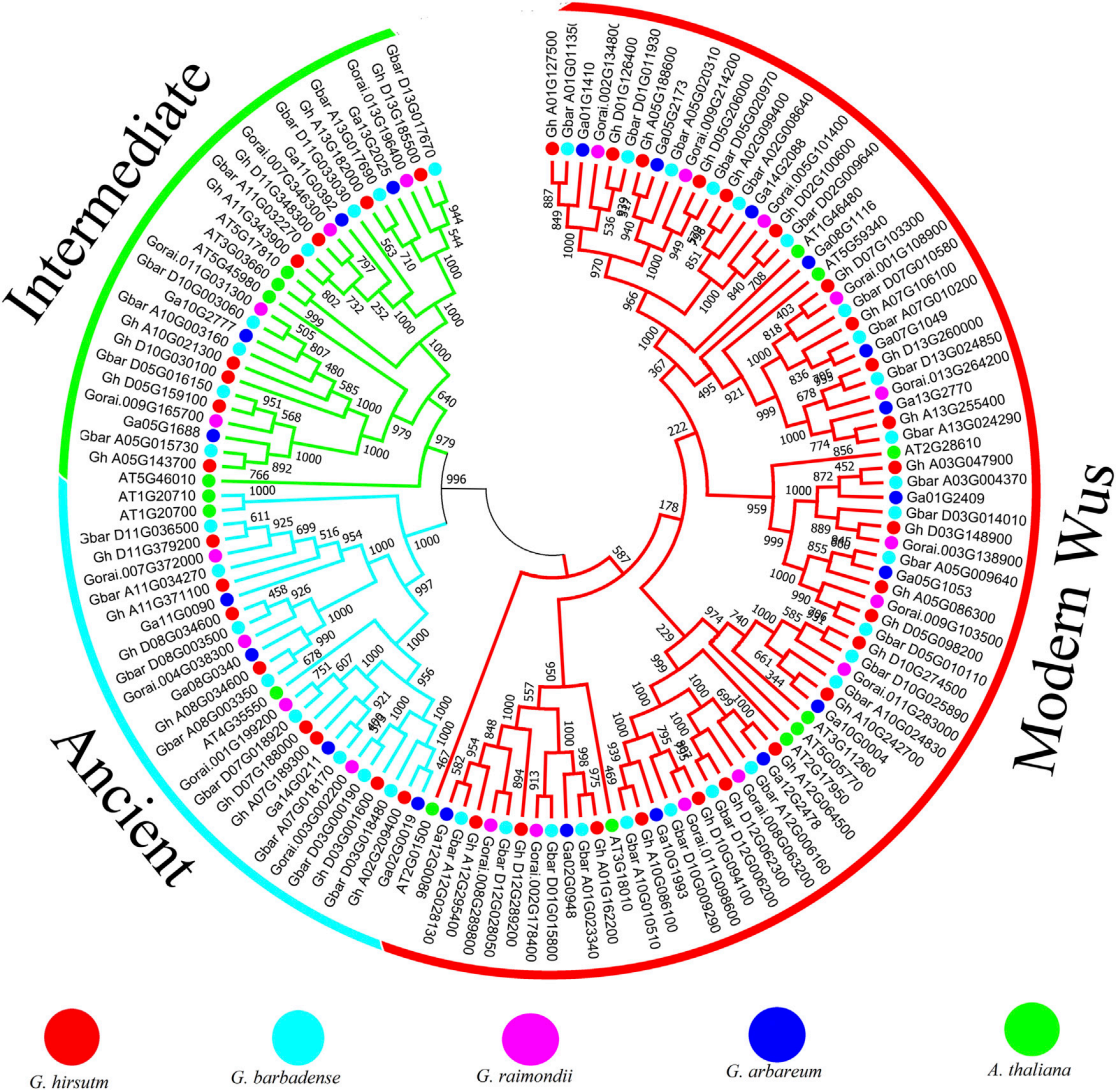
Phylogenetic tree of *WOX* genes in *Gossypium* species and *Arabidopsis*.

The phylogenetic tree result shows that all the *WOX* genes could be classified into three clades, which is consistent with the previous result in other species (Figure 2). Eight, eight, four, and four *WOX* genes from *G. hirsutum*, *G. barbadense*, *G. arboreum*, and *G. raimondii* were classified into the intermediate clade. Eight, eight, four, and four *WOX* genes from *G. hirsutum*, *G. barbadense*, *G. arboreum*, and *G. raimondii* were classified into the ancient clade. The modern/WUS clade includes 23, 24, 12, and 13 *WOX* genes from *G. hirsutum*, *G. barbadense*, *G. arboreum*, and *G. raimondii*. From the gene structure analysis result, we can find that most *WOX* genes have three or four exons, and those genes classified into the same clade tend to have similar gene structures (Supplementary Figure S1). The patterns of motifs were studied to elucidate the structural evolution of WOX proteins. Multiple motifs were identified, and results revealed that motif one was conserved in four species, while motif two was conserved in *G. barbadense* and *G. raimondii* (Supplementary Figure S2). We further found that motif four was conserved in the genes that were classified into the intermediate clade in all four species, and motif two and six were conserved in the genes that were classified into the ancient clade in *G. hirsutum* and *G. barbadense*. In contrast, motif three was conserved in the genes classified into the ancient clade in both *G. arboreum* and *G. raimondii*.

### Expression analysis of *WOX* gene in *G. hirsutum* cultivar ZM24

**FIGURE 3:**
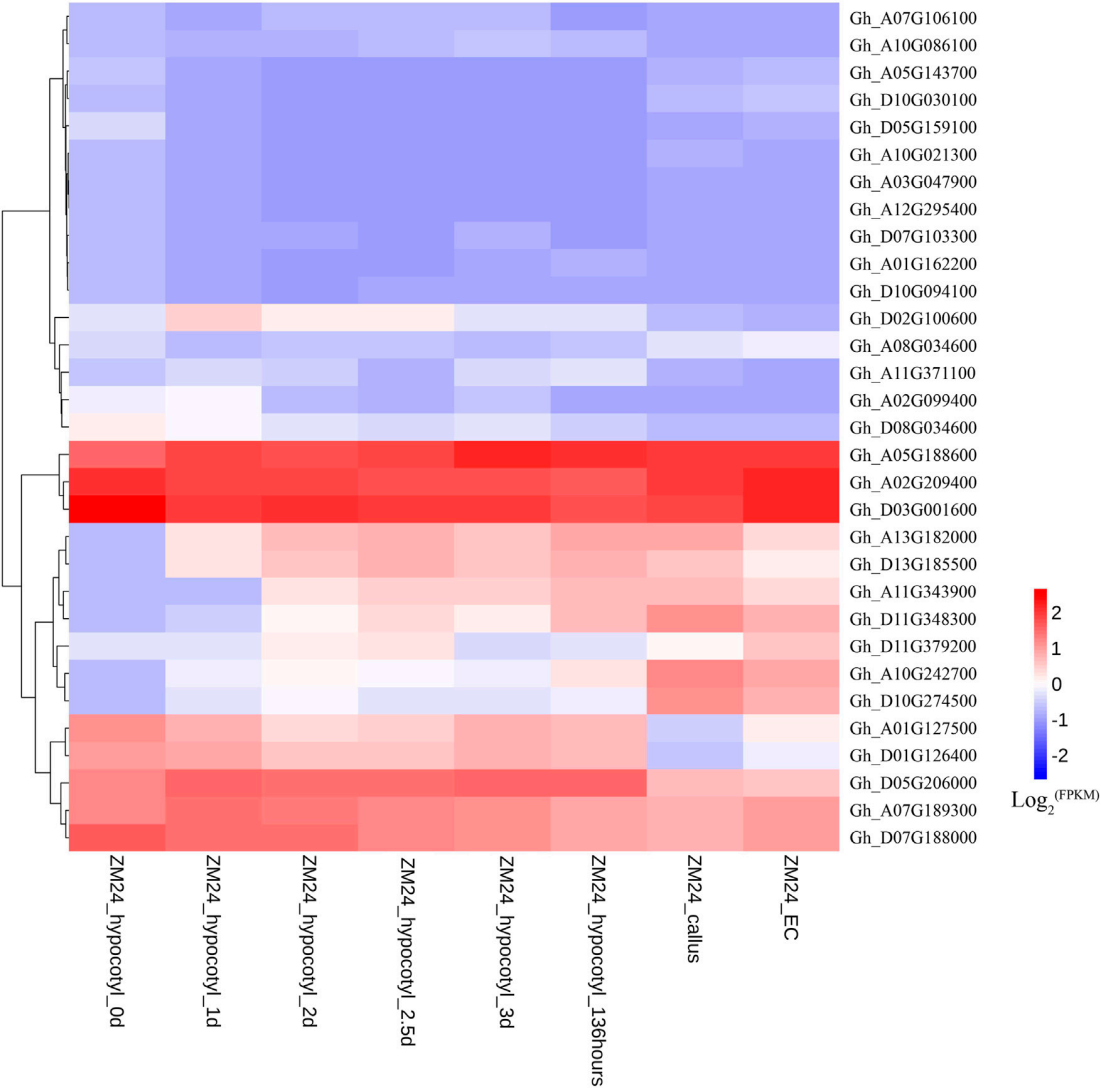
RNA-seq data analysis of *WOX* genes in germinating hypocotyls, callus, and embryonic callus (EC) in ZM24.

Expression patterns of *WOX* genes in *G. hirsutum* variety ZM24 were analyzed, and eight genes that did not have expression in all these tissues are not shown in Figure 3. Sixteen genes have higher expression levels and 15 genes have lower expression levels in germinating hypocotyls, callus, and embryonic callus. We further analyzed expressions of these 16 *WOX* genes in callus in four different varieties (Figure 3).

### Callus induction rate and expression of *WOX* genes in shoot tip, hypocotyl, and cotyledon-induced callus in four cotton varieties

**FIGURE 4:**
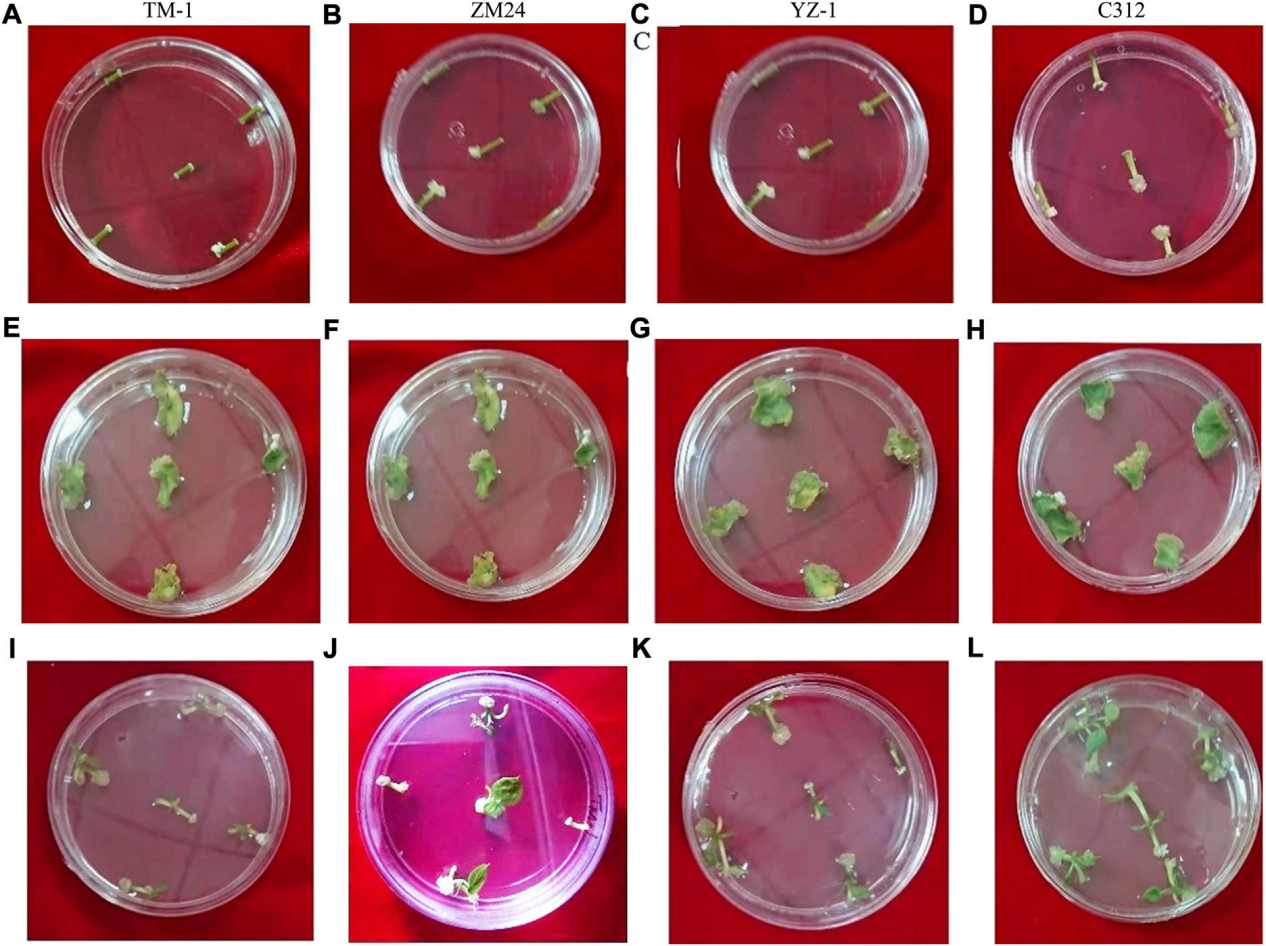
Callus induction rate in *G. hirsutum*. **(A)** 1-day hypocotyl callus induction for TM-1, **(B)** 2-day hypocotyl callus induction for ZM24, **(C)** 2.5-day hypocotyl callus induction for YZ-1, **(D)** 3-day hypocotyl callus induction for C312, **(E)** 1-day cotyledon callus induction for TM-1, **(F)** 2-days’ cotyledons callus induction for ZM24, **(G)** 2.5-day cotyledon callus induction for YZ-1, **(H)** 3-day cotyledon callus induction for C312, **(I)** 1-day shoot tip callus induction for TM-1, **(J)** 2-day shoot tip callus induction for ZM24, **(K)** 2.5-day shoot tip callus induction for YZ-1, and **(L)** 3-day shoot tip callus induction for C312.

Callus induction rate analysis was carried out on hypocotyl, cotyledon, and shoot tips (Figure 4). For 1-day, 2-day, 2.5-day, and 3-day callus induction, shoot tip and hypocotyl have much higher callus induction rates than cotyledon. Subculturing of calli after 2 weeks into the MS medium increased the size of the calli in different tissues of *G. hirsutum*. Callus with different textures, sizes, and appearances was fully observed among four cotton varieties (Supplementary Figure S3).

**FIGURE 5:**
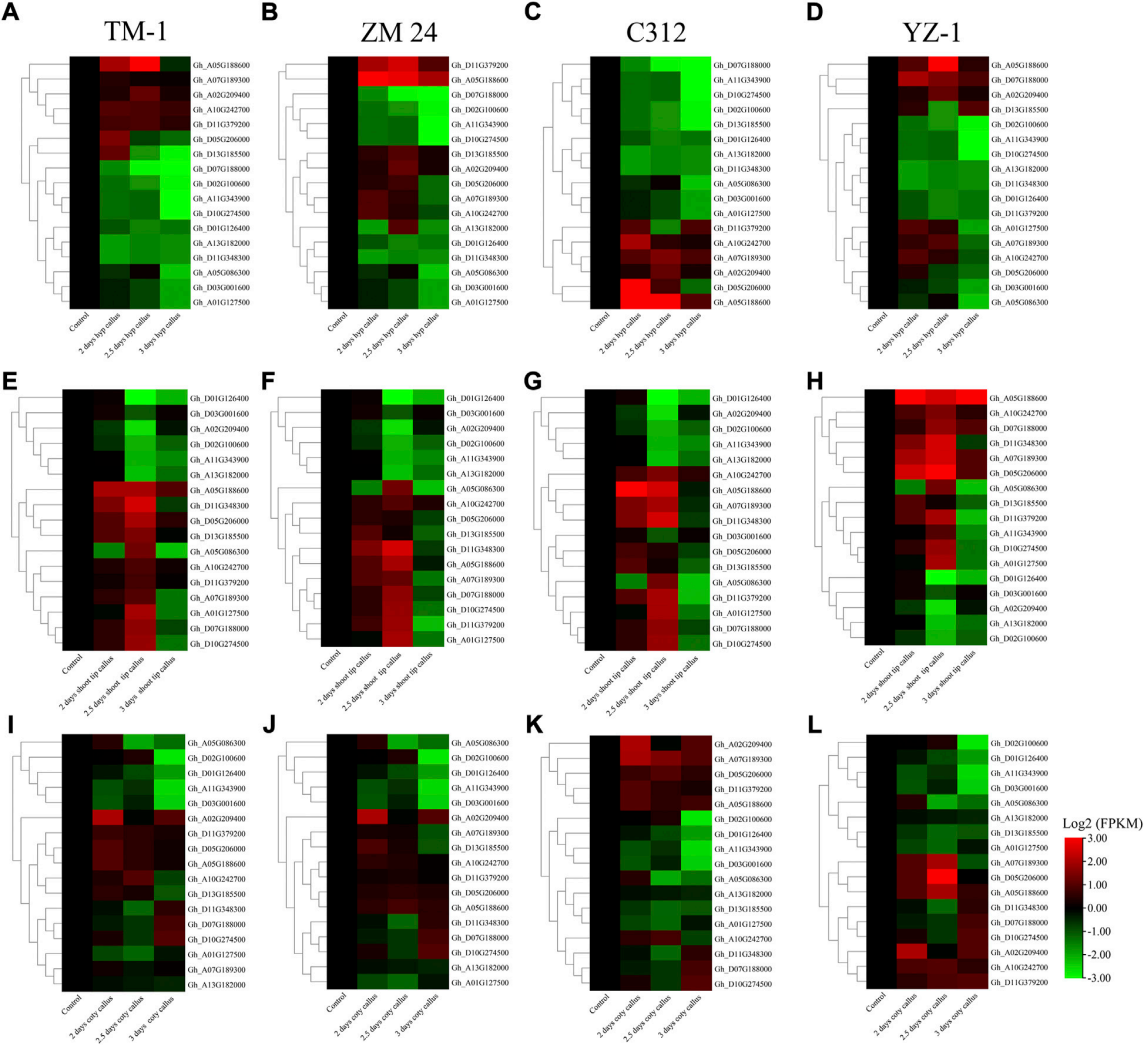
Expression profile of 16 *WOX* genes in shoot tip, hypocotyls, and cotyledon-induced callus in four different varieties. **(A)** Callus induced from hypocotyls in TM-1, **(B)** callus induced from hypocotyls in ZM24, **(C)** callus induced from hypocotyls in YZ-1, **(D)** callus induced from hypocotyls in C312, **(E)** callus induced from the shoot in TM-1, **(F)** callus induced from the shoot in ZM24, **(G)** callus induced from the shoot in YZ-1, **(H)** callus induced from the shoot in C312, **(I)** callus induced from cotyledon in TM-1, **(J)** callus induced from cotyledon in ZM24, **(K)** callus induced from cotyledon in YZ-1, and **(L)** callus induced from cotyledon in C312.

To further analyze the expression profile of the above 16 *WOX* genes in callus, we examined the expression of selected genes in three types of callus induced from shoot tip, hypocotyl, and cotyledon in four varieties. The result showed that most genes were up-regulated in all three types of callus in four varieties, indicating that *WOX* genes play vital roles in callus induction (Figure 5). First, more genes have higher expression in callus induced from shoot tip than callus induced from hypocotyls and cotyledon. Second, *WOX* genes have similar expression patterns in callus induced from the same explants. In four varieties, *Gh_A05G188600* was up-regulated in both three types of callus.

### Network interaction prediction of WOX proteins

From the constructed protein interaction network, we can know that ten proteins have high predicted interaction levels with WOX4, including Clavata3, Homeobox8 (HB-8), Clavata3/Embryo Surrounding Region-Related (CLE) 44, and CLE41 (Supplementary Figure S4). The structure of the vascular meristem during secondary growth is influenced by a component of the Tracheary Element Differentiation Inhibitory Factor (TDIF)-TDIF Receptor (TDR)-WOX4 signaling pathway. Phloem intercalated with xylum (PYX) is a leucine-rich repeat receptor-like protein kinase that acts with CLE41 and CLE44. ATHB-8 is a homeobox-leucine zipper protein that may have a role in controlling vascular development, which shares 31.8% of identity with the WOX4 protein and is thought to promote precambial and cambial cell differentiation.

### *GhWOX4_A01* transcriptional activation assay

Three vectors including pGBKT7- *GhWOX4_A01*, pGBKT7, and pGADT7-largeT + pGBKT7-p53 were transformed into the AH109 yeast. All transformants could grow in SD/-Trp medium and turned blue in the SD/-Trp + X-α-gal medium but did not grow in the SD/-Trp-Ade-His medium (Supplementary Figure S5). This result indicated that *GhWOX4_A01* has no activation activity.

### *GhWOX4_A01* silenced plants showed significant sensitivity to drought

**FIGURE 6:**
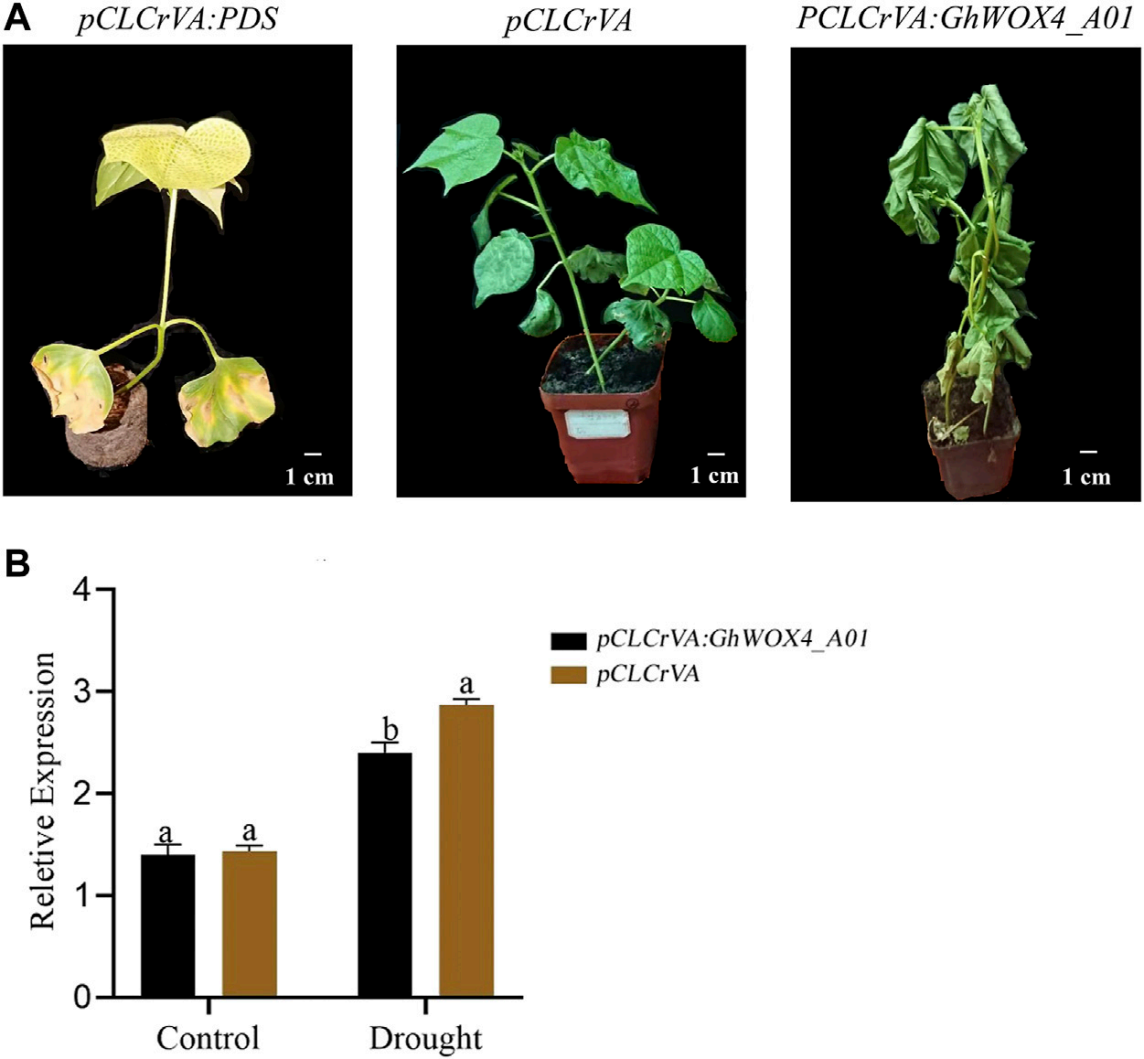
Phenotypic characterization of *GhWOX4-A01* silenced plants. **(A)** Negative control, positive control, and *GhWOX4_A01* silenced plants and **(B)** qRT-PCR analysis of *GhWOX4_A01* in control and silenced plants under control conditions and after 10 days drought treatment. Different letters show significant differences at *p* < 0.05.

**FIGURE 7:**
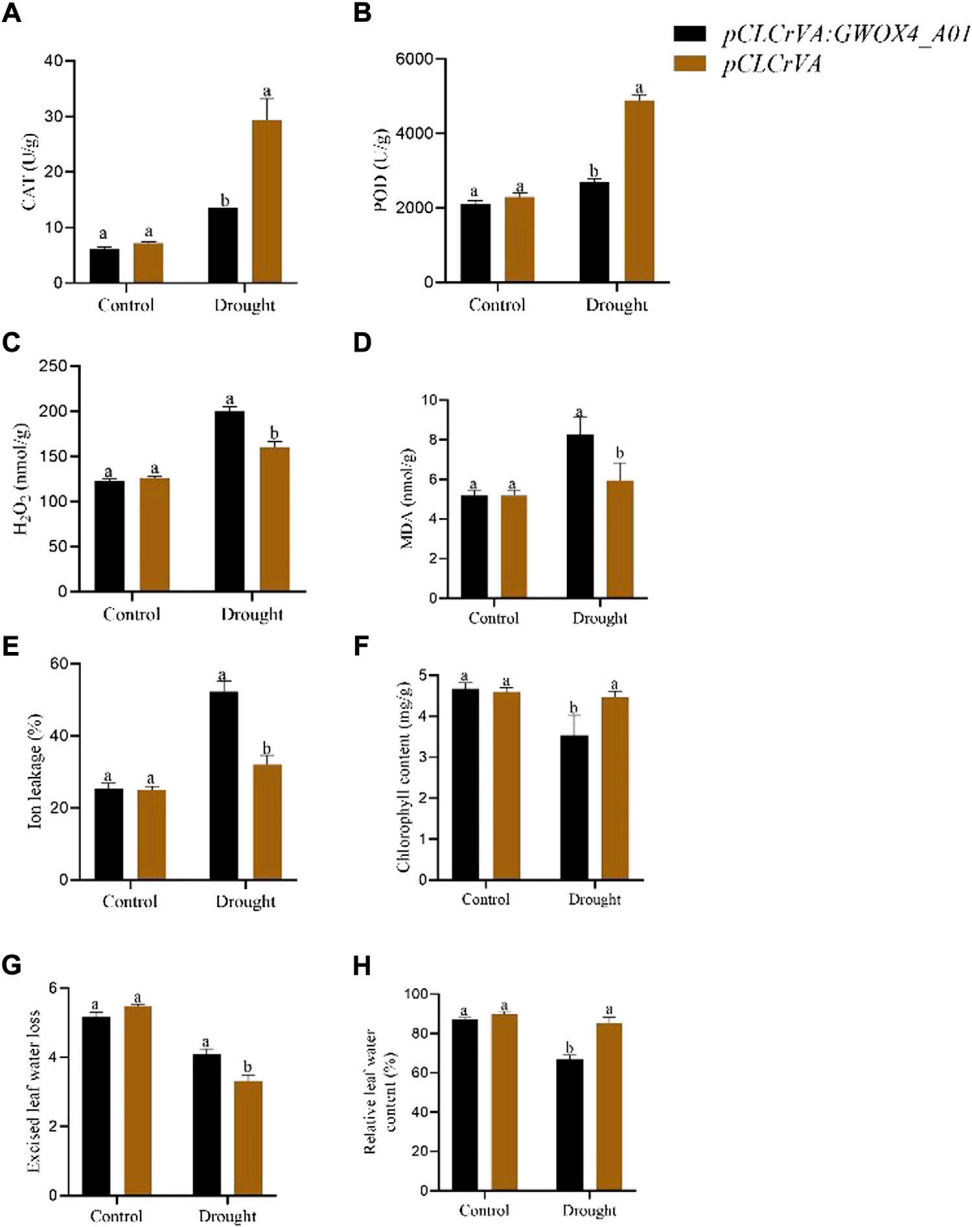
Physiological traits and enzyme activity in control and *GhWOX4*-*A01* silenced plants under control conditions and after 10 days of drought treatments. **(A)** CAT, **(B)** POD, **(C)** H_2_O_2_, **(D)** MDA, **(E)** ion leakage percent, **(F)** chlorophyll content, **(G)** extracted leaf water lost, and **(H)** relative leaf water content percent. Each experiment was carried out three times. The error bar represents the standard deviation of the three biological replicates. The significant difference was indicated by different letters at *p* < 0.05.

The function of the *GhWOX4_A01* (*Gh_A01G127500*) in drought tolerance was investigated using the VIGS approach. The indicator *pCLCrVA: PDS* showed an albino color, while the control plant had a normal color without visible change. The silenced plants *pCLCrVA: GhWOX4_A01* showed complete shrinkage of the leaves, indicating total silencing of the gene (Figure 6A). qRT-PCR was used to analyze the expression level of *GhWOX4_A01* (Figure 6B). Under control and drought circumstances, the concentrations of antioxidant enzymes (CAT and POD) and oxidants (MDA and H_2_O_2_) were determined in both control and silenced plants. Under control conditions, both the antioxidant enzymes and oxidants did not have a significant difference between control and silenced plants. After drought treatment, the oxidant concentrations were significantly higher, but the antioxidant enzymes were significantly lower in silenced plants than in respective controls (Figures 7A–D).

The effects of *GhWOX4_A01* on plant physiological changes during drought stress were investigated. Important parameters were investigated, including ion leakage, excised leaf water loss, chlorophyll concentration, and relative leaf water content. Compared to the control plant, the relative electrolyte leakage level of the infected plant (*pCLCrVA: GhWOX4_ A01*) increased by about 15% (Figure 7E). The chlorophyll content of infected plants decreased compared to control plants. However, in terms of excised leaf water loss, the infected plants increased significantly more than the control plants (Figures 7F,G). Under drought conditions, the relative leaf water content of the infected plant drops to 65%, compared to 86% for the control (Figure 7H).

## Discussion

Cotton is one economically important crop for the textile industry. *WOX* gene family is highly conserved in plants. Previous studies reported that *WOX* genes play important roles in stem cell regulation, embryo patterning, and abiotic stress (Breuninger et al., 2008; Dolzblasz et al., 2016; Minh-Thu et al., 2018). Although the function of *WOX* genes has been well studied in the model plant *Arabidopsis*, the specific roles of *WOX* genes in callus induction, regeneration, and stress response are not yet well understood. In this study, we found 39 *WOX* genes in *G. hirsutum*, which was different from previous research (Yang et al., 2017). For example, Gh_D01G1463 cannot be found based on the genome sequence used in this study, and we found this gene has a high identity with the intergenic sequence between Gh_D01G167200 and Gh_D01G167300 (Supplementary Table S3). Furthermore, the previous study could not find two genes in our study (Gh_A11G371100 and Gh_D12G289200). We believe all these differences were due to different versions of the *G. hirsutum* genome*.* Gene loss usually occurs due to hybridization and chromosome doubling (Paterson et al., 2004). In this study, we determined gene loss by comparing the number of *WOX* genes in *G. hirsutum* (39) and *G. barbadense* (40) with the sum of the gene numbers of its two progenitors (41). The orthologous gene of *Gorai.002G178400* was lost in the Dt subgenome of *G. hirsutum.* The orthologous gene of *Ga08G1116* was lost in *G. raimondii*, *G. hirsutum*, and *G. barbadense*, and this gene was also the minimum gene that only encoded 66 amino acids*.* Previous research has shown that the *WOX* gene family can be divided into the ancient, intermediate, and WUS clades. In this study, eight, eight, and 23 *WOX* genes from *G. hirsutum* were classified into the intermediate, ancient, and modern/WUS clades, respectively, which is consistent with previous research that the WUS clade was much higher than either the intermediate or ancient clade (Deveaux et al., 2008). Both the gene structure and conserved motif analysis showed that *WOX* genes classified into the same group tend to have similar structures and motifs, which strongly supported the close evolutionary relationships among the *WOX* genes within each subfamily.

The *WOX* genes have been pivotal in organ formation, embryo patterning, and stem cell maintenance (Deveaux et al., 2008; Nardmann and Werr 2012). RNA-seq and qRT-PCR data analysis showed that most genes are expressed in germinating hypocotyls, callus, and embryonic callus. Our result indicated that these genes might be involved in callus induction and regeneration. Previous studies showed that most *WOX* genes in rice and soybean were responsive to abiotic stress (Cheng et al., 2014; Hao et al., 2019). Overexpression of *OsWOX13* resulted in drought resistance in rice (Minh-Thuet al., 2018). Abiotic stresses such as drought stress inhibit plant growth, including reducing the photosynthetic rate and electrolyte pressure (Magwanga et al., 2019; López et al., 2020). To determine if *GhWOX4_A01* was involved in drought stress response in cotton, we have silenced its expression by VIGS and measured relative water content, ion leakage, excised water loss, and chlorophyll content in both the silenced plants and the negative control under normal and drought stress conditions. The finding revealed that the silenced plants showed more water loss and ion leakage, and the chlorophyll content was lower than that of the control under drought stress conditions. Reactive oxygen species (ROS) are by-products of cellular metabolism, which are usually produced under stress conditions. However, the plant developed effective mechanisms, including antioxidant molecules and antioxidant enzymes, to minimize the damage caused by ROS (Atkinson and Urwin, 2012). In this study, the knockdown plant (*pCLCrVA: GhWOX4_A01*) has higher MDA and H_2_O_2_ content than the control plants. CAT and POD levels were lower in the treated plants (*pCLCrVA: GhWOX4_A01*) than in the control plants (*pCLCrAVA*). These results indicated that *GhWOX4_A01* might increase cotton tolerance to drought by maintaining homeostasis of ROS.

## Conclusion

This study provides a genomic framework for the cotton *WOX* gene family, and 39, 40, 21, and 20 *WOX* genes were identified in *G. hirsutum*, *G. barbadense*, *G. arboreum*, and *G. raimondii*. Moreover, gene loss events occurred in the *WOX* gene family in *G. hirsutum* and *G. barbadense* during the hybridization of two progenitors. Phylogenic analysis showed that all the *WOX* genes could be classified into the ancient, intermediate, and WUS clades. The *WOX* gene family in cotton was highly conserved at the DNA and protein levels. Most of the *WOX* gene expression level was up-regulated in germinating hypocotyls and callus. *GhWOX4_A01* was highly expressed in embryonic callus than in regular callus. The silenced plants (*pCLCrVA: GhWOX4_A01*) have accumulated more oxidants and were more sensitive to drought treatment. Taken together, our results can enhance our understanding of the role of the *WOX* gene family in tissue regeneration and abiotic stress, and provide a reference for future molecular analysis.

## Data Availability

The original contributions presented in the study are included in the article/Supplementary Material; further inquiries can be directed to the corresponding authors.
