# Supplementary figures and images for: Characterization of WOX genes revealed drought tolerance, callus induction, and tissue regeneration in Gossypium hirsutum
Source: Front Genet. 2022 Oct 12;13:928055. doi: 10.3389/fgene.2022.928055 (PMC9597092; doi:10.3389/fgene.2022.928055)

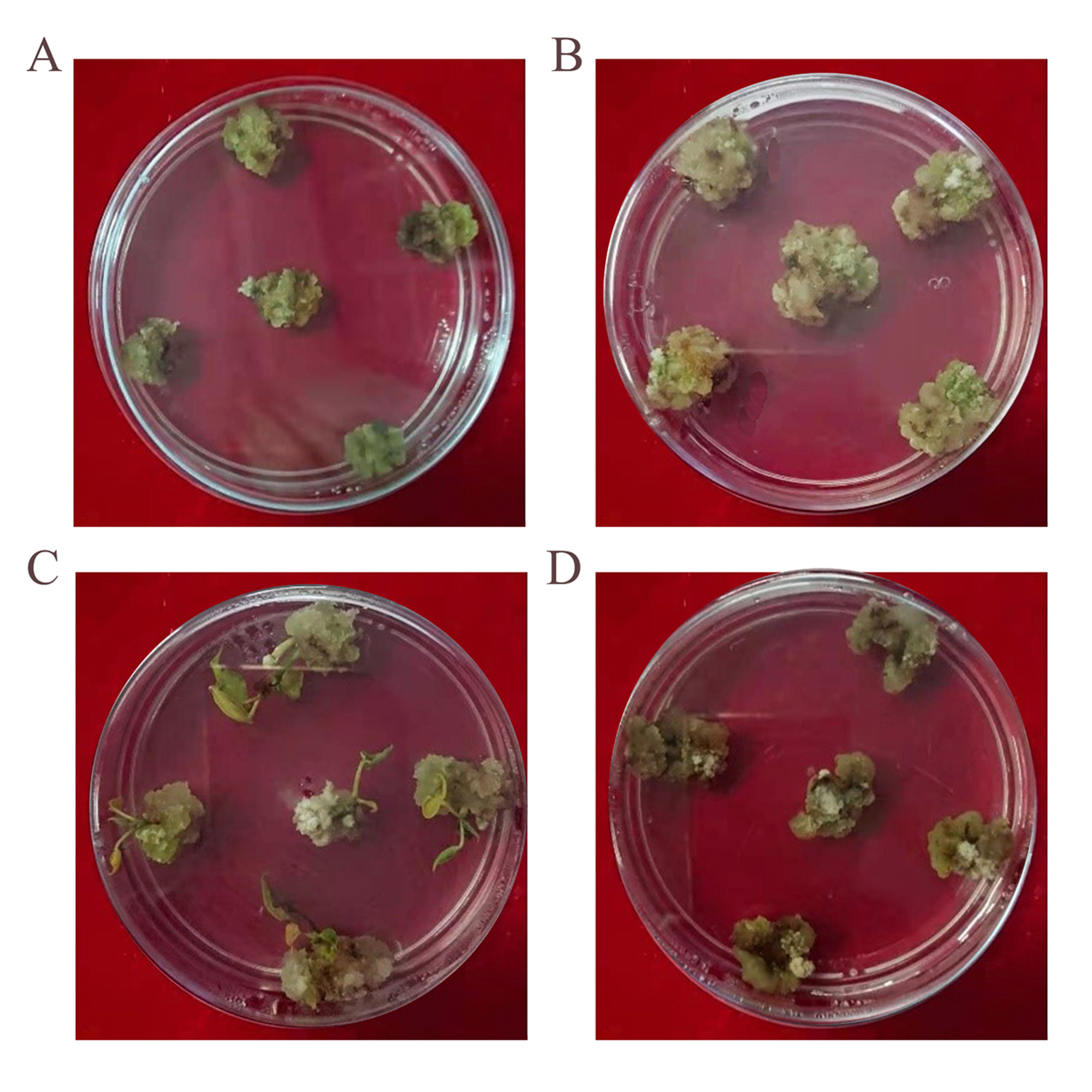

Supplement: Supplementary file 1 [file Image3.JPEG]

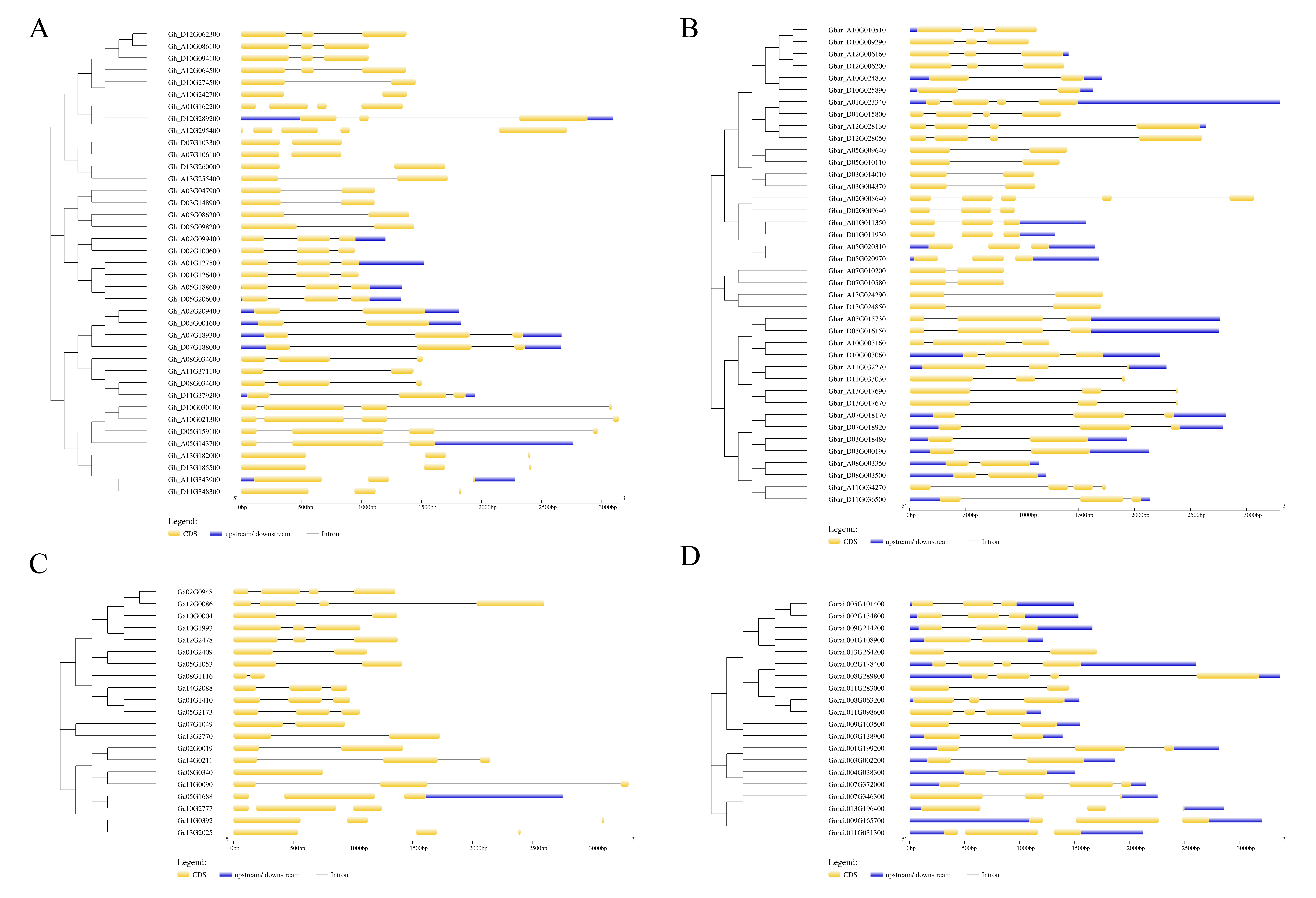

Supplement: Supplementary file 3 [file Image1.JPEG]

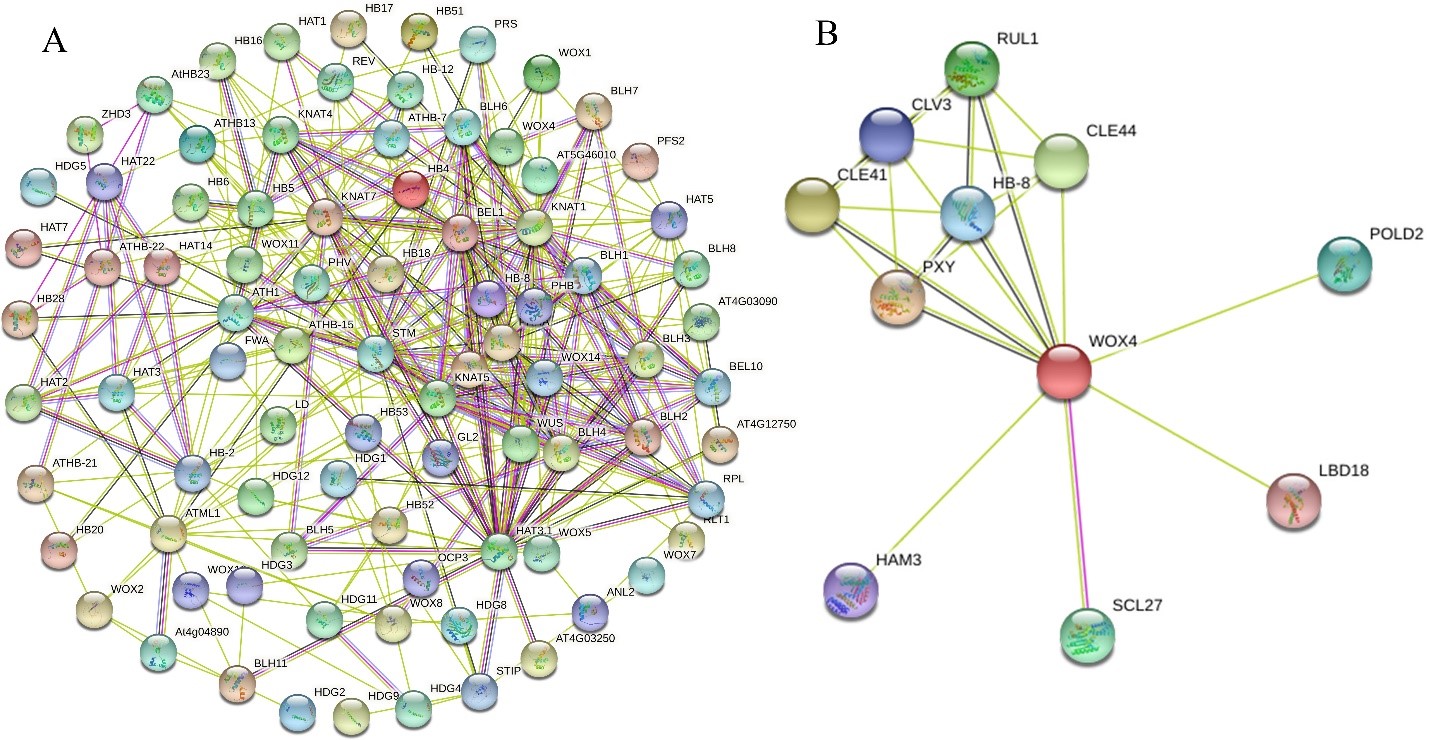

Supplement: Supplementary file 4 [file Image4.JPEG]

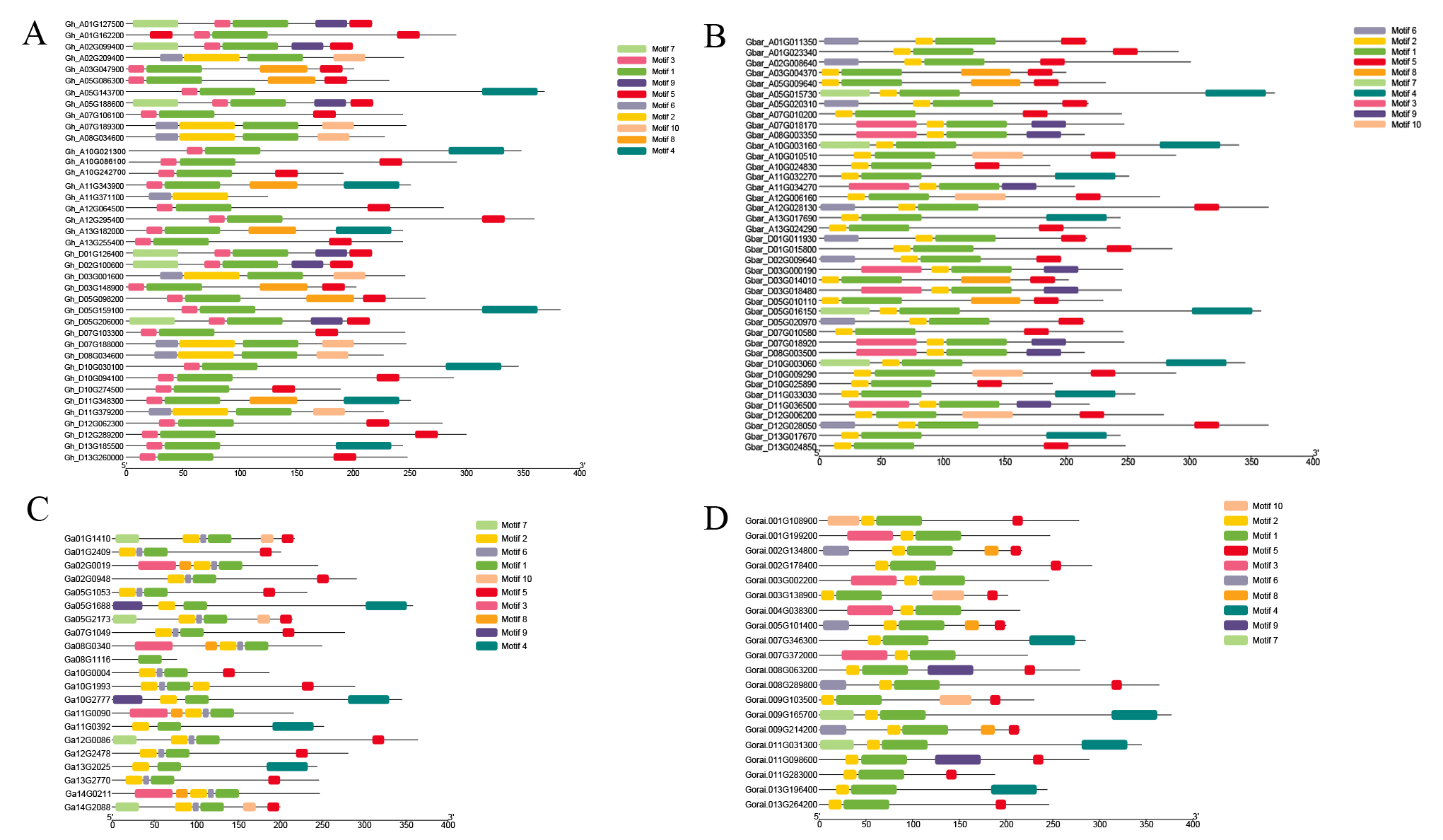

Supplement: Supplementary file 5 [file Image2.JPEG]

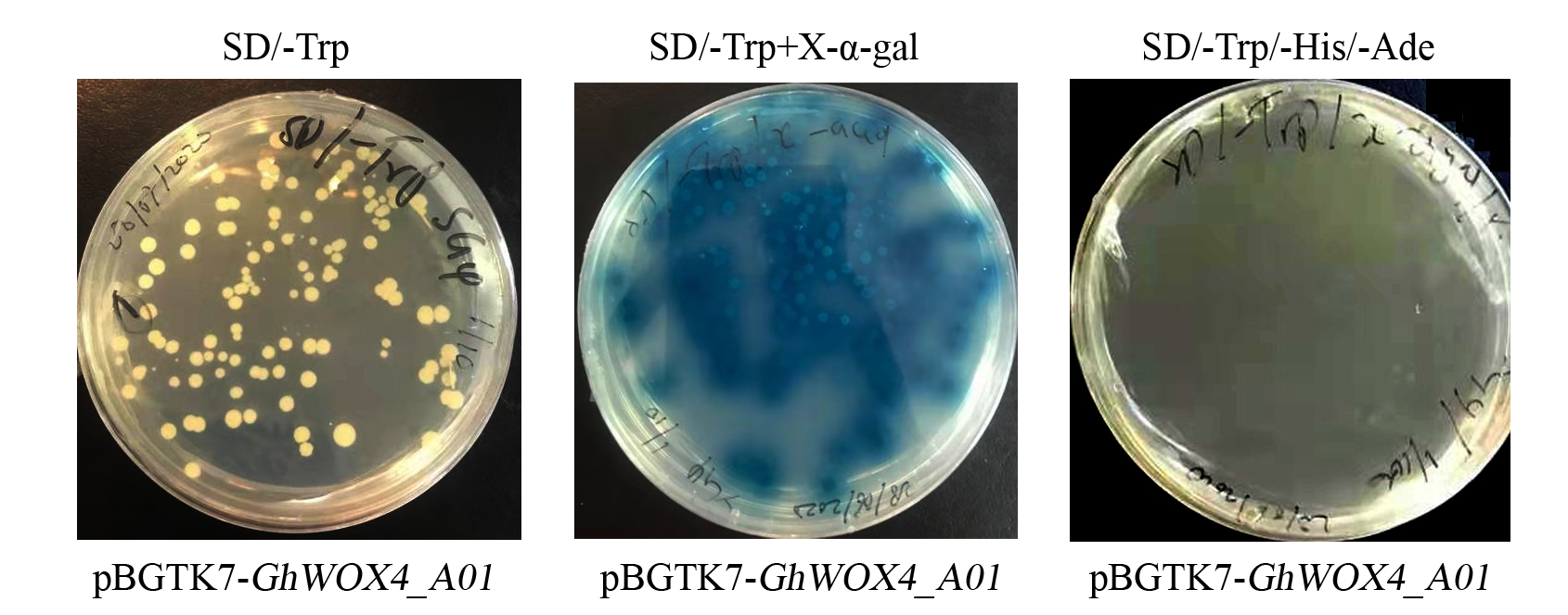

Supplement: Supplementary file 6 [file Image5.JPEG]
