# Supplementary material for: Characterization of WOX genes revealed drought tolerance, callus induction, and tissue regeneration in Gossypium hirsutum
Source: Front Genet. 2022 Oct 12;13:928055. doi: 10.3389/fgene.2022.928055 (PMC9597092; doi:10.3389/fgene.2022.928055)
Supplement: Supplementary file 7 [file Table2.DOCX]

| **Primer Name** | **Primer sequence (5'-3')** | **Purpose** |
| --- | --- | --- |
| Gh_ A01G127500-VIGS-L | ATGCCTGCAGACTAGTGCCCTCTTGCTCCAAAGCTA | VIGS |
| Gh_ A01G127500-VIGS-R | AGACCTAGGGGCGCGCCTCTCCCGTGCTTTGTGGTTT | VIGS |
| Gh_ A01G127500-qRT-L | GAGGAAGTGCAGAAGCTGGT | qRT-PCR |
| Gh_ A01G127500-qRT-R | TGCAATGGGAAGAGCTCCAG | qRT-PCR |
| Gh_ A01G127500-YH-L | CCGCTGCAGGTCGACGGATCCATGGGAAACATGAAGATG | Yeast hybrid |
| Gh_ A01G127500-YH-R | ATGGCCATGGAGGCCGAATTCTCATCTGCTTTCCGGGTGCA | Yeast hybrid |
| Gh_A07G189300-qRT-L  Gh_A07G189300-qRT-R  Gh_A10G242700-qRT-L  Gh_A10G242700-qRT-R  Gh_A11G343900-qRT-L  Gh_A11G343900-qRT-R  Gh_A13G182000-qRT-L  Gh_A13G182000-qRT-R  Gh_D01G126400-qRT-L  Gh_D01G126400-qRT-R  Gh_D02G100600-qRT-L  Gh_D02G100600-qRT-R  Gh_D03G001600-qRT-L  Gh_D03G001600-qRT-R  Gh_D05G206000-qRT-L  Gh_D05G206000-qRT-R  Gh_D07G188000-qRT-L  Gh_D07G188000-qRT-R  Gh_D10G274500-qRT-L  Gh_D10G274500-qRT-R  Gh_D11G348300-qRT-L  Gh_D11G348300-qRT-R  Gh_D11G379200-qRT-L  Gh_D11G379200-qRT-R  Gh_D13G185500-qRT-L  Gh_D13G185500-qRT-R  Gh_A02G209400-qRT-L  Gh_A02G209400-qRT-R  Gh_A05G086300-qRT-L  Gh_A05G086300-qRT-R  Gh_A05G188600-qRT-L  Gh_A05G188600-qRT-R | \| GCAACTGGGGTCATCATCCA \| \| --- \| \| AGATCTTCAGCCCTTGGTGC \| \| CAACAAGATTTCTTCTTCGAAACGG \| \| TCGTTTGCATGGAATCTCAGC \| \| AGAAGTTCCAAGGGGTCCAC \| \| CACCGTGGAGCAAACTCTCA \| \| GGTTCCGTTGGGGATGCTAA \| \| TTGTTCTGAACCTGCTGCGA \| \| GCTGGCTTGTGGATTTTGGG \| \| TGTAGCTTTGGGGCAAGAGG \| \| TACGGCAAGATCGAAGGCAA \| \| TGGACCATCCTCCCTAGTGTT \| \| GAGGGTCAGGGTCAACATCA \| \| GGTCATAAATGCAGCTTCCCC \| \| TTCTGGGAGCACGAACCATC \| \| GGAGGTGTGCTGCTGTAACT \| \| GCAACTGGGGTCATCATCCA \| \| AGATCTTCAGCCCTTGGTGC \| \| AAGGGTTGTCGGGGTTTTGT \| \| AGGGTTCCATCTCCCACACT \| \| TCCAAAACCGACGTTCGAGG \| \| ACCGCCGTTACCGAGTATTT \| \| TCAGCCAATGCTGAACCTGA \| \| TCAATCCCTGCAAAAAGAACTGAT \| \| GGTTCCGTTGGGGATGCTAA \| \| CACCGTATACTGCTTGGGCT \| \| CTCTCCGGAAGCAGATTGCC \| \| GTGCCCACCCGATGTCATTA \| \| GTTATAACCCTGCCTCCGCC \| \| TGGCCTTGTTCTTGTCCATTTT \| \| TTCTGGGAGCACGAACCATC \| \| GGAGGTGTGCTGCTGTAACT \| | qRT-PCR  qRT-PCR  qRT-PCR  qRT-PCR  qRT-PCR  qRT-PCR  qRT-PCR  qRT-PCR  qRT-PCR  qRT-PCR  qRT-PCR  qRT-PCR  qRT-PCR  qRT-PCR  qRT-PCR  qRT-PCR  qRT-PCR  qRT-PCR  qRT-PCR  qRT-PCR  qRT-PCR  qRT-PCR  qRT-PCR  qRT-PCR  qRT-PCR  qRT-PCR  qRT-PCR  qRT-PCR  qRT-PCR  qRT-PCR  qRT-PCR  qRT-PCR |

Table S2. All primers used in this research.
